# Impact of Intestinal Microbiota on Growth Performance of Suckling and Weaned Piglets

**DOI:** 10.1128/spectrum.03744-22

**Published:** 2023-04-06

**Authors:** Md Rayhan Mahmud, Ching Jian, Md Karim Uddin, Mirja Huhtinen, Anne Salonen, Olli Peltoniemi, Heli Venhoranta, Claudio Oliviero

**Affiliations:** a Department of Production Animal Medicine, Faculty of Veterinary Medicine, University of Helsinki, Helsinki, Finland; b Human Microbiome Research Program, Faculty of Medicine, University of Helsinki, Helsinki, Finland; c Orion Corporation, Orion Pharma, R&D, Espoo, Finland; University of Saskatchewan

**Keywords:** pig, gut microbiota, growth, fiber degrading bacteria, weaning, SCFA, NGS sequencing

## Abstract

Small-scale studies investigating the relationship between pigs' intestinal microbiota and growth performance have generated inconsistent results. We hypothesized that on farms under favorable environmental conditions (e.g., promoting sow nest-building behavior, high colostrum production, low incidence of diseases and minimal use of antimicrobials), the piglet gut microbiota may develop toward a population that promotes growth and reduces pathogenic bacteria. Using 16S rRNA gene amplicon sequencing, we sampled and profiled the fecal microbiota from 170 individual piglets throughout suckling and postweaning periods (in total 670 samples) to track gut microbiota development and its potential association with growth. During the suckling period, the dominant genera were *Lactobacillus* and *Bacteroides*, the latter being gradually replaced by *Clostridium sensu scricto* 1 as piglets aged. The gut microbiota during the nursery stage, not the suckling period, predicted the average daily growth (ADG) of piglets. The relative abundances of SCFA-producing genera, in particular *Faecalibacterium, Megasphaera, Mitsuokella*, and *Subdoligranulum*, significantly correlated with high ADG of weaned piglets. In addition, the succession of the gut microbiota in high-ADG piglets occurred faster and stabilized sooner upon weaning, whereas the gut microbiota of low-ADG piglets continued to mature after weaning. Overall, our findings suggest that weaning is the major driver of gut microbiota variation in piglets with different levels of overall growth performance. This calls for further research to verify if promotion of specific gut microbiota, identified here at weaning transition, is beneficial for piglet growth.

**IMPORTANCE** The relationship between pigs' intestinal microbiota and growth performance is of great importance for improving piglets’ health and reducing antimicrobial use. We found that gut microbiota variation is significantly associated with growth during weaning and the early nursery period. Importantly, transitions toward a mature gut microbiota enriched with fiber-degrading bacteria mostly complete upon weaning in piglets with better growth. Postponing the weaning age may therefore favor the development of fiber degrading gut bacteria, conferring the necessary capacity to digest and harvest solid postweaning feed. The bacterial taxa associated with piglet growth identified herein hold potential to improve piglet growth and health.

## INTRODUCTION

The gut microbiota has critical functions in the maintenance of animal health (1). The pig gut harbors a diverse microbial community, containing 10^10^ to 10^11^ microorganisms per gram of intestinal material (2), which act as a key regulator in nutrition, digestion, disease resistance, and the synthesis of vitamins and other metabolites (3, 4). Early gut colonizers in pigs are crucial in establishing persistent microbial community patterns that affect health and growth performance later in life (5, 6), and are shaped by various factors, such as dietary changes, maternal milk, probiotics and prebiotic supplements, and in-feed antibiotics (5, 7).

The suckling period is a significant stage for neonatal development, providing immunity and energy sources from colostrum and milk (8). Moreover, colostrum and milk potentially include bacteria and prebiotic substances (e.g., oligosaccharides) that aid maturation of the neonatal gut (3, 9). However, increased litter size poses a challenge to colostrum intake of neonate piglets, resulting in subsequent impaired passive immunity and retarded intestinal development (10, 11). Such conditions can increase the incidence of pathogenic bacteria proliferation in the gut, thereby increasing the use of antimicrobials (12). Moreover, the impaired development of the intestinal microbiota during the suckling period can be further deviated during weaning due to stressors such as diet change and animal regrouping (13).

Piglets are weaned and separated from the mother at the end of the suckling period (around 3 to 4 weeks old), and they have to rely only on solid feed. Weaning affects the intestinal architecture and is a critical period for gut microbiota development in young pigs (3, 4). The gut microbiota of newborn pigs undergoes rapid ecological succession (5), shifting its composition substantially during weaning (14). Early studies demonstrated that weaning transition reduced the abundance of *Lactobacillus* and gut microbiota diversity, whereas *Clostridium*, *Prevotella* spp., and Proteobacteriaceae (including Escherichia coli) significantly increased (15, 16). Establishing a healthy gut microbiota during weaning is critical because piglets have an undeveloped immune system and rely on sow milk to prevent opportunistic pathogen colonization and overgrowth (17).

Gaukroger et al. recently found that the piglet gut microbiota is associated with their age, birth weight, and consequent average daily growth (ADG) (18). They demonstrated a positive association of gut microbiota richness with birthweight at 21, 27, 32, and 56 days in piglets. They noted that piglets with the most increased ADG according to the average of their birth weight class had higher relative abundances of *Lactobacillus*, *Ruminococcaceae* UCG-005 and unclassified *Prevotellaceae* at 4, 8, and 14 days of age, respectively (18). In another study, the piglets with higher preweaning ADG had the fecal microbiota enriched with *Lactobacillus*, *Flavonifractor*, *Barnesiella*, *Gemmiger*, *Faecalibacterium*, *Roseburia*, and *Anaerophaga* at 1 week of age. In contrast, slow-growing piglets with low preweaning ADG had more *Desulfovibrio*, *Acidaminobacter*, *Dethiosulfatibacter*, *Fastiduisipila*, *Ruminnococcus*, and *Anaerotruncus* at 1 week of age (19). A French study of piglets from 16 different farms showed that the highest relative growth rate during the first 3 weeks after weaning had a higher relative abundance of *Bacteroidetes*, a lower relative abundance of *Proteobacteria*; the fast-growing piglets also showed a greater increase in *Prevotella*, *Coprococcus*, and *Lachnospira* in the postweaning period (20). While existing studies suggest the potential importance of the piglet gut microbiota in animal health, often there is no consensus regarding the correlation between the gut microbiota and growth at different stages. On the other hand, the gut microbiota development in piglets is influenced by the sow’s health status and, therefore, the maternal gut microbiota, environment, colostrum intake, colostrum composition and the feeding practices (7, 19). There are farms where all these external inputs are more favorably oriented (e.g., sows are provided with large amounts of bedding material to promote nest-building behavior, produce more colostrum, piglets’ suckling period is longer, they have low incidence of diseases, and minimal use of antimicrobials). In contrast, other farms have less favorable conditions (e.g., sows are given a limited amount of bedding material, piglets’ suckling period length is conventional, sows have high incidence of diseases, and there is an average or above average use of antimicrobials). Comparison between these different farms in terms of the piglet gut microbiota from birth until weaning may allow identification of specific gut microbes associated with better growth and health.

We hypothesized that under favorable environmental conditions, such as the ones described above, piglets' gut microbiota may develop toward a population that promotes growth and reduces pathogenic bacteria. A better understanding of the gut microbiota dynamics during suckling and weaning in a well-characterized, large animal cohort will provide important information for the development of intervention strategies aimed at improving piglet growth. Thus, we profiled the fecal microbiota of piglets from birth to the postweaning period using Illumina sequencing of the 16S rRNA gene amplicons to identify potential associations between piglet growth (ADG) and gut microbes.

## RESULTS

### Development of the piglet gut microbiota.

**FIG 1:**
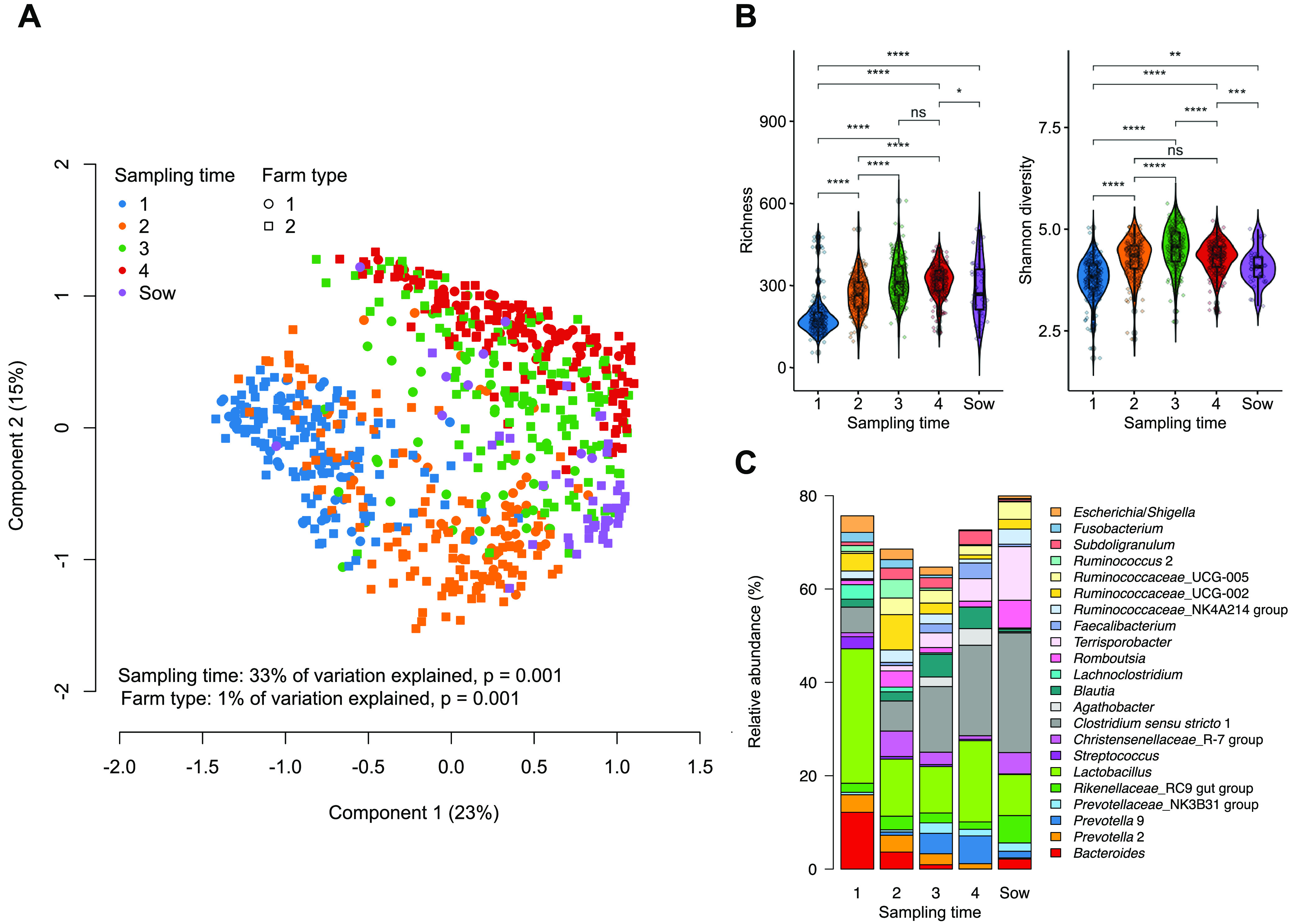
Longitudinal gut microbiota profiles from all piglets and sows’ gut microbiota profiles (A) Principal coordinate analysis (PCoA) plot of microbiota variation based on the Bray-Curtis dissimilarity matrix. Sampling time explained 33% of the microbiota variation, while farm type explained only 1% of the microbiota variation (*P* = 0.001, PERMANOVA, [farm type 1 = farm 1; farm type 2 = farms 2, 3, and 4]). (B) Violin plots (a combination of the box plot with a kernel density plot) showing microbiota α-diversity (richness and Shannon diversity) at each sampling time in comparison to the sow gut microbiota. The center line denotes the median, the boxes cover the 25th and 75th percentiles, and the whiskers extend to the most extreme data point, which is no more than 1.5 times the length of the box away from the box. Points outside the whiskers represent outlier samples. Significance was calculated using the Wilcoxon rank-sum test. ****, *P* < 0.0001; ***, *P* < 0.001; **, *P* < 0.01; *, *P* < 0.05; “ns” *P* > 0.05. (C) Stacked bar plots showing the average relative abundance of dominant bacterial genera (>1%).

The composition of the gut microbiota changed markedly over sampling times, visualized by strong age-driven clustering of the samples on the PCoA based on the Bray-Curtis dissimilarity matrix (Fig. 1A). Microbiota α-diversity, assessed by richness and Shannon diversity, gradually increased and peaked at sampling time 3 (Fig. 1B). The α-diversity was significantly lower in sows compared to postweaning piglets (Fig. 1B). In terms of individual bacterial genera, there was no statistically significant difference between the piglets belonging to farm type 1 and 2 at any given sampling time (FDR-*P* > 0.05; Table S6). However, the relative abundances of *Christensenellaceae*_R-7 group, *Ruminococcaceae*_UCG-002, Treponema 2, *Romboutsia*, and *Turicibacter* were significantly more abundant in the sows from farm type 2 (FDR-*P* < 0.05; Table S6). The analyses were subsequently done using the pooled data from all four farms. The changes in the mean relative abundances of dominant bacterial genera (>1% mean relative abundance) are shown in Fig. 1C. Specifically, *Prevotella* 9, *Agathobacter*, *Faecalibacterium*, *Terrisprobacter*, and *Clostridium sensu stricto* 1 increased in relative abundance after weaning (5 to 6 weeks and 8 to 9 weeks aged piglets), while E. coli, Streptococcus, and *Bacteroides* showed the opposite trend. The expansion of acetate-producing *Terrisporobacter* and butyrate-producing *Clostridium sensu stricto* 1 appeared to continue into adulthood, as the two fiber-degraders dominated the sow gut microbiota (Fig. 1C). The relative abundance of *Lactobacillus* declined after sampling time 1 and subsequently increased at sampling time 4. Moreover, we noted a large decrease in *Ruminococcaceae_*UCG-002 between sampling time points 2 and 3.

### Importance of microbiota covariates at various developmental stages.

**FIG 2:**
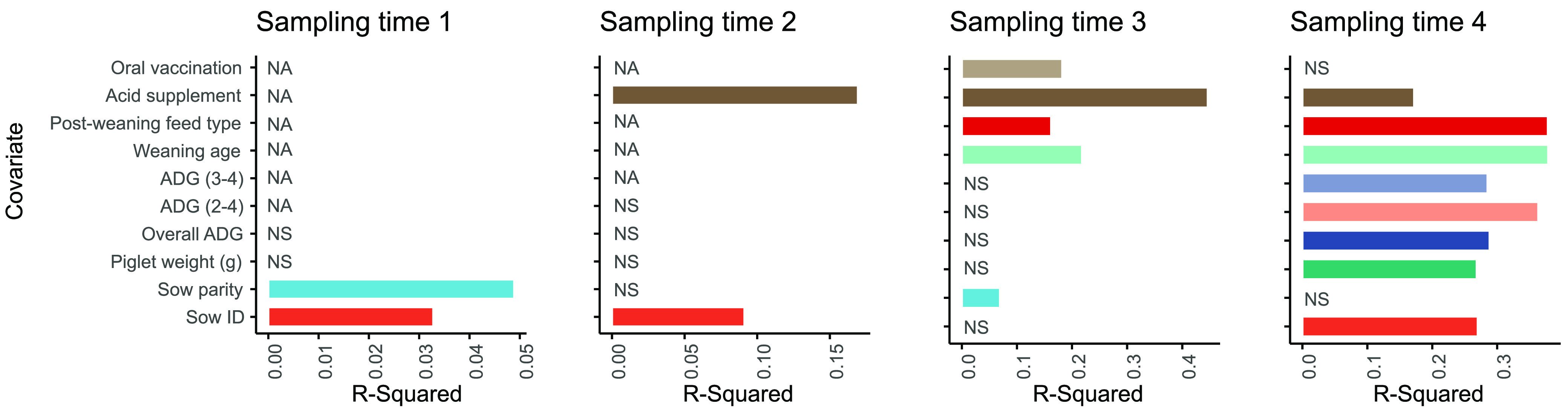
Explained variance of select microbiota covariates at various sampling times modeled by *envfit*. Horizontal bars show the degree of variance (R-squared) explained by each covariate in the model using microbiota profiles at the genus level. The covariates not applicable for the given sampling time are denoted with NA. Nonsignificant covariates are denoted with NS.

We next explored the effects of environmental, maternal, and host variables on the piglet gut microbiota variation by *envfit*. Fig. 2 shows the identified associations between available covariates and the gut microbiota at different sampling times. For the neonates (sampling time 1, 5 to 9 days), maternal factors, i.e., sow parity and sow ID, were strongly associated with microbiota variation as expected; at sampling time 2 (3 to 4 weeks), sow ID was still significantly associated with microbiota variation in addition to acid supplement in water. After weaning, the effect of external variables became more evident. At sampling time 3 (5 to 6 weeks old piglets), oral vaccination against E. coli, acid supplement, feed type after weaning, weaning age (days), and sow parity were found to be significantly associated with microbiota variation. Postweaning feed type, average daily growth, piglet weight, and weaning age (days) were the significant covariates associated with microbiota variation at sampling time 4 (8 to 9 weeks old piglets).

### Associations between the gut microbiota and piglet growth.

**FIG 3:**
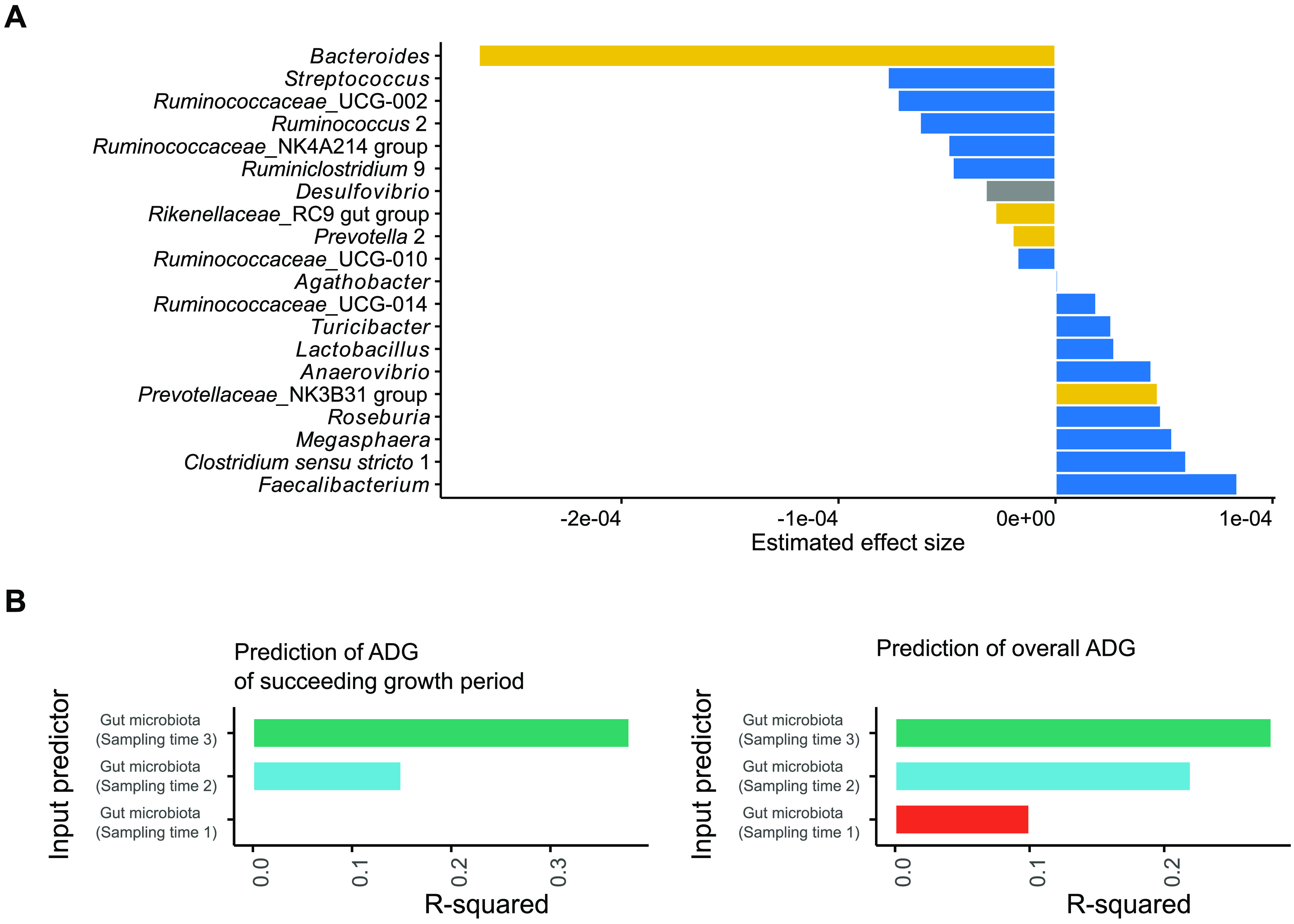
Associations between the gut microbiota and overall piglet growth or growth during specific periods. (A) Bar plots showing significant associations (FDR-*P* < 0.05) between individual bacterial genera and overall ADG. Bacterial genera are colored according to their respective phyla (Bacteroidetes in yellow, Proteobacteria in gray, and Firmicutes in blue). (B) Bar plots showing the predictive power (R-squared) of the gut microbiota at sampling point 1, 2, and 3 for the ADG of its succeeding period (i.e., the growth between sampling point 1 to 2, 2 to 3, and 3 to 4) and the overall ADG.

Having established a significant relationship between the overall gut microbiota and piglet age, we attempted to identify individual bacterial genera significantly associated with piglet ADG. Across the entire follow-up time, *Faecalibacterium* had the strongest positive correlation with growth, while *Bacteroides* had the strongest negative correlation (Fig. 3A).

To evaluate whether piglet growth can be predicted by gut microbiota composition, the ADG between each sampling time (i.e., the growth between sampling point 1 to 2, 2 to 3, and 3 to 4) was predicted using the gut microbiota data collected at the beginning of the corresponding sampling time (i.e., sampling times 1, 2, and 3, respectively) in a random forest regression model (Fig. 3B). The gut microbiota at sampling time 3 explained a large proportion of variance (ca. 38%) in the ADG between sampling point 3 to 4, while only 15% of the variance in the ADG between sampling point 2 to 3 could be explained by the gut microbiota composition at sampling time 2. The baseline gut microbiota (sampling time 1) failed to predict the ADG of its succeeding growth period. Similar findings were evident when the overall ADG was predicted. These findings indicate that the gut microbiota and piglet growth became strongly associated during and after weaning.

### Developmental trajectory and maturity of the gut microbiota and their association with piglet growth.

**FIG 4:**
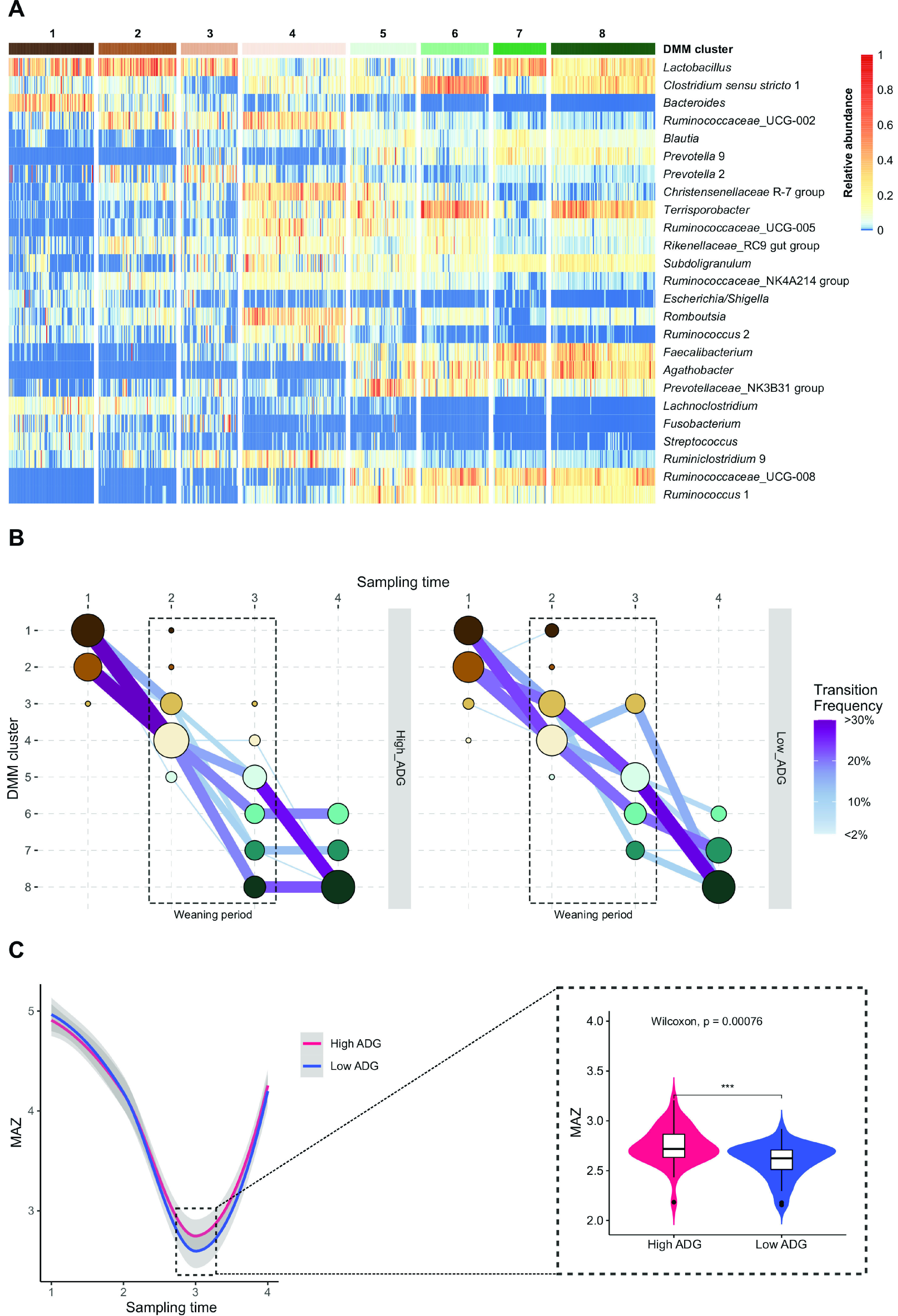
Microbiota development and maturity and their associations with piglet growth. (A) Heat map showing the relative abundances of the 25 most dominant bacterial genera per Dirichlet multinomial mixtures (DMM) cluster. DMM clustering using the entire piglet data set formed eight distinct clusters. (B) Transitions between DMM clusters throughout sampling time points in the high (N = 157) or low (N = 158) ADG group. The sizes of nodes and edges are scaled by the number of included samples; nodes are colored according to DMM clusters and edges by the transition frequency. Transition frequency was determined by dividing the number of transitions toward a given DMM cluster by the total number of transitions within each time window. Single edges (transitions) are not shown. (C) Microbiota-by-age Z-scores (MAZ), reflecting microbiota maturity, throughout four sampling times in the high-ADG (black line) and low-ADG (blue line) group as modeled using the Loess regression. Gray areas represent the 95% confidence intervals.

We hypothesized that gut microbiota development differs between fast-growing (high ADG) and slow-growing (low ADG) piglets. To visualize microbiota development, we first identified the samples with similar microbiota composition in the entire cohort of piglets by Dirichlet multinomial mixtures (DMM) clustering (Fig. 4A), and then visualized the transitions between DMM clusters throughout sampling time points based on ADG class (Fig. 4B). When examining the developmental trajectory of the gut microbiota in the high and low-ADG groups (Fig. 4B), both ADG groups similarly transitioned from clusters 1 and 2 (dominated by *Lactobacillus*) to clusters 3 and 4 during sampling time 1 to 2. Upon weaning (sampling time 3), most piglets in the high-ADG group transitioned to clusters 5 to 8, whereas the low-ADG group transitioned to clusters 3, 5, 6, and 7. No piglets in the low-ADG group reached cluster 8 after weaning. Notably, the transition in the high-ADG group was characterized by a stabilization phase between sampling time 3 to 4, suggesting that the gut microbiota largely reached maturity after weaning. In contrast, the progression toward clusters 6 to 8 continued during sampling time 3 to 4 in the low-ADG group. Of note, clusters 6 to 8 were the most dominant at the last sampling time, being characterized by the near complete disappearance of *Bacteroides* and *Lachnoclostridium* and the dominance of fiber-degrading *Faecalibacterium*, *Agathobacter*, and *Ruminococcaceae_*UCG-008. To evaluate microbiota maturity, microbiota age was estimated by training a machine-learning algorithm on the microbiota composition of a data set of known biological age, and thus the age of samples was predicted based on microbiota composition. A microbiota-by-age Z-score (MAZ) was then calculated for each sample that reflects microbiota maturity. The faster microbiota maturation in the high-ADG group, suggested by the transition of DMM clusters mentioned previously, was supported by a significantly higher MAZ upon weaning compared with the low-ADG group (*P* < 0.05; Fig. 4C).

### Differentially abundant bacterial genera during weaning based on piglet growth.

**FIG 5:**
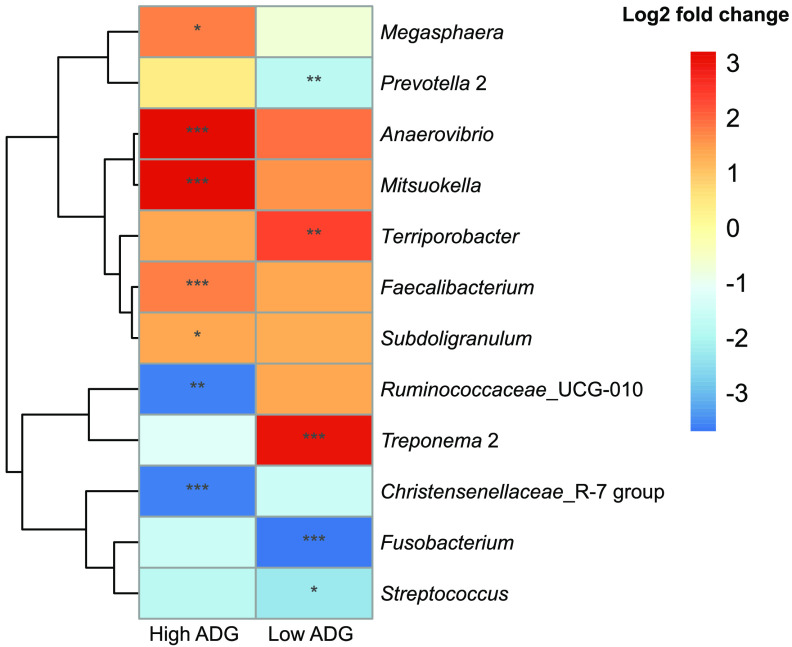
ADG group-specific enrichment or depletion of bacterial genera in the gut microbiota during weaning. Heat map showing the 14 bacterial genera with increased or decreased relative abundances, calculated by log_2_ fold change, during weaning (sampling time 2 to 3) statistically significant only in high- or low-ADG group (FDR-*P* < 0.05, log_2_ fold change > 1). ***, FDR-*P* < 0.001; **, FDR-*P* < 0.01; *, FDR-*P* < 0.05.

Because the gut microbiota development during weaning differed considerably between the high and low-ADG groups, we subsequently identified individual bacterial genera that significantly increased or decreased in relative abundance during weaning (cut-off FDR-*P* < 0.05, log_2_ fold change > 1), specifically in the high- and low-ADG groups (Fig. 5). The relative abundances of five genera (*Anaerovibrio*, *Faecalibacterium*, *Megasphaera*, *Mitsuokell*, and *Subdoligranulum*) significantly increased while *Christensenellaceae* R-7 group and *Ruminococcaceae*_UCG-010 significantly decreased during weaning (sampling time 2 to 3) only in the high-ADG group. On the other hand, the relative abundances of Treponema 2 and *Terrisprobacter* were significantly elevated while *Fusobacterium*, *Prevotella* 2, and Streptococcus depleted in low-ADG piglets during weaning.

## DISCUSSION

In this study, we characterized the development of the gut microbiota longitudinally from birth to weaning and identified associations between growth performance and the gut microbiota in a large cohort of piglets. Our results showed that: (i) The preweaning gut microbiota of piglets was dominated by bacteria specialized in utilizing milk oligosaccharides. The postweaning gut microbiota was characterized by the dominance of fiber-degrading genera. (ii) Piglet growth measured by ADG was more strongly associated with the postweaning gut microbiota than that of suckling age. (iii) Fast-growing piglets largely reached microbiota maturity upon weaning, whereas slow-growing piglets continued their microbiota maturation after weaning.

Overall, we established an increasing trend in gut microbiota α-diversity in the growing piglets until the age of 6 weeks, which is consistent with previous studies (21–23). Thereafter, a decrease in microbiota α-diversity in 8- to 9-week-old piglets was observed, similar to the finding by Hu et al. (24). Microbiota α-diversity in sows was significantly lower than that in postweaning piglets. The sow gut microbiota, although similarly dominated by fiber degraders, was somewhat different from that of piglets, suggesting the possible long-term cumulative effect of aging, diet, rearing, and environment conditions on the pig gut microbiota. In our study, the high relative abundances of the bacterial genera E. coli, *Lactobacillus*, Streptococcus, and *Bacteroides* were gradually replaced by strictly anaerobic fermenters, consistent with previous studies (5, 13). Taken together, our and others’ findings demonstrated the consistent succession of gut microbes in piglets and suggest that the gut microbial diversity is saturated in piglets at 5 to 6 weeks of age (23, 25).

We recorded that *Bacteroides*, E. coli, and *Lactobacillus* predominated the gut microbiota during the first week after birth, in accordance with the study by Luo et al. (26). The nutrient up-take capabilities of *Bacteroides* and *Lactobacillus* from milk oligosaccharides and host-derived glycans make them the predominant genera in preweaning piglets’ gut (5, 26, 27). *Lactobacillus* plays an important role in maintaining animal health during early life (27, 28) and gradually decreases its abundance approaching the weaning period (15, 27). Facultative anaerobes such as lactobacilli and E. coli colonize the gut right after birth. Their high abundances create an oxygen-deprived environment in the intestine during weaning and favor the expansion of obligate anaerobes (29) that are typically specialized in degrading plant-derived complex polysaccharides. The relative abundance of *Lactobacillus* increased again when the piglets were 8 to 9 weeks old, likely promoted by postweaning feed rich in fiber as some species of *Lactobacillus* are known polysaccharide degraders (30). After weaning, piglets are usually exposed to solid feed containing various cereals, and different vegetables and traces of animal protein (31). The faster transition toward the postweaning intestinal microbiota equipped with the capacity for complex carbohydrate utilization endows piglets with the improved ability to acquire nutrition from the feed (23). Recent studies reported that fiber-degrading genera were significantly enriched in piglets upon weaning (23, 24), which we corroborated in this study where the relative abundances of *Faecalibacterium*, *Agathobacter*, and *Prevotella* 9 (dominated by Prevotella copri) increased upon weaning. Pigs lack the enzymes to degrade fibrous compounds, and thus the gut microbiota is essential for degrading fiber by fermentation that produces metabolites that maintain energy homeostasis (32–34). *Faecalibacterium* and *Agathobacter* ferment indigestible fibers to produce butyrate, one of the major short-chain fatty acids (SCFAs). Butyrate possesses anti-inflammatory properties and is beneficial for the development of porcine gut health (35). The high relative abundance of *Faecalibacterium* in the piglet gut microbiota before weaning could prevent postweaning dysbiosis potentially by countering inflammation (36, 37). The reduction of *Faecalibacterium* in the gut microbiota has been documented in several dysbiosis-related diseases in humans (38). *Prevotella*, another important feature of the postweaning gut microbiota, has been associated with positive outcomes in pig production, including growth performance and immune response in many studies (39).

We identified several novel factors significantly associated with the piglet gut microbiota at different sampling times. Notably, maternal factors were mostly associated with the gut microbiota variation before weaning, while feed type, environmental, and growth-related variables were important after weaning. This supports the finding that the development of the gut microbiota after weaning may be related to several farm management factors. The fact that the postweaning microbiota was most strongly associated with piglet growth highlights the importance to improve postweaning gut health of piglets on commercial farms. Moreover, considering the potential impact of the sow gut microbiota on preweaning piglets’ microbiota, improving gut health in sows may benefit their offspring. We had a particular interest in comparing farms with standard commercial characteristics to one special farm with enhanced welfare conditions as an exploratory analysis. Somewhat surprisingly, the differences in the overall gut microbiota and the relative abundance of bacterial taxa between the two types of farm did not reach statistical significance despite that farm type 1 had the best growth after weaning (Table 1). While the commercial farms in our study shared important environment and rearing conditions, they differed in other known (e.g., postweaning feed type) and unknown (or unmeasured) factors that may influence the gut microbiota. For example, a recent study found that levels of disinfection in farrowing facilities can impact the piglet gut microbiota from birth to weaning (40). By comparing these commercial farms as a group to farm type 1, we inevitably introduced the influence of the intractable factors that cannot be adequately adjusted statistically. Therefore, future studies collecting a host of detailed environment and rearing-related factors are warranted to investigate the impact of farm-specific conditions on the piglet gut microbiota while controlling for feed type. Nevertheless, we did observe more nominally significant and statistically significant differences in the relative abundance of bacterial taxa in the postweaning piglets and sows, respectively, between two types of farms. This again suggests that the living environment has more influence on the piglet gut microbiota after weaning than before as recently reported by Luise et al. (20).

**TABLE 1:** Different intervals of average daily growth (ADG) by farm

Farm type	Farm type 1	Farm type 2		
Farms	Farm 1 (*n* = 172)	Farm 2 (*n* = 158)	Farm 3 (*n* = 174)	Farm 4 (*n* = 168)
ADG^*a*^ (1 to 2)	262.08 ± 4.71	298.61 ± 4.92	302.59 ± 5.09	308.07 ± 5.03^*b*^
ADG (2 to 3)	247.22 ± 5.45^*b*^	216.95 ± 5.19	239.43 ± 5.54	138.63 ± 3.41
ADG (3 to 4)	602.16 ± 7.92^*b*^	485.73 ± 10.91	267.27 ± 6.62	414.83 ± 8.72
ADG (1 to 4)	420.67 ± 5.42^*b*^	362.41 ± 6.37	271.34 ± 4.22	291.96 ± 4.24

a ADG in g/day were given as mean ±SEM.

b *P* < 0.05.

The link between the gut microbiota and growth performance of pigs has been at the center of research in recent years. Our data indicate that the postweaning microbiota is more important than that of suckling age regarding its association with growth performance. This is logical considering the previously discussed role of microbial fermentation in energy metabolism. However, we cannot exclude the potential role of initial colonization and/or early programming by the microbiota during the suckling period. Recent research showed that birth weight influenced piglets’ gut bacteria from days 7 to 21 (41). Low-birth-weight piglets have significantly higher postnatal mortality, slow development, and poor carcass quality (26, 41, 42). Lu et al. showed that the gut microbiota before weaning was not significantly associated with body fat or ADG of pigs, but a significant association was found later at 15 weeks of age (43). In addition, Maltecca et al. demonstrated that the gut microbiota of pigs may be utilized to predict growth and body composition metrics, especially fatness attributes, when sampled later in life (44). This finding was recapitulated in our study. In general, however, few studies have examined whether the gut microbiota differs between poor growing piglets and better growing piglets and whether there is any role of gut microbiota in the growth performance of piglets. Our study design allowed us to address this question and we demonstrated apparent differences in microbiota development between high-growth and low-growth piglets upon weaning. We found that the gut microbiota in high-ADG piglets matured faster and reached the stabilization phase after weaning, while the gut microbiota in low-ADG piglets continued to develop after weaning. Similar findings have been observed in weaning and ADG studies in humans (45).

During the critical time window of weaning, we found significant increases in the relative abundances of *Faecalibacterium*, *Mitsuokella*, *Anaerovibrio*, *Megasphaera*, and *Subdoligranulum* in the high-ADG piglets. *Megasphaera* and *Mitsuokella* utilize certain substrates, including lactate to produce SCFAs, which are associated with improved gut health in piglets by stimulating growth and proliferation of small intestinal cells (46, 47). Yang et al. showed that *Mitsuokella* positively correlated with body weight and average daily growth in piglets (47). On the other hand, *Subdoligranulum*, abundant in Duroc pigs (5), is a newly discovered bacterial genus and a butyrate producer with potential in improving host metabolic health (48).

### Limitations.

Although adjusted statistically when applicable, the analyses where piglets from different farms were pooled may be confounded by postweaning feed type and latent farm-level differences in the present study, and thus, the results must be interpreted with caution. Our study design was chosen partly because we had a particular interest in comparing farms with standard commercial characteristics to a farm with enhanced welfare conditions and a much longer suckling period. While we failed to establish a statistically significant difference in the gut microbiota between the two types of farm, detailed environment and rearing conditions should be collected and investigated in future studies controlling for feed type. The choice of sampling only female piglets was to avoid the bias in growth performance and possible stress of male piglets at the time of first sampling. Studies on sex difference in gut microbiota of young piglets are currently scarce. Hence, it remains to be tested whether our results can be generalized to male piglets. Finally, although causality cannot be established from our findings, the present study is an important primer for filling the knowledge gaps regarding the timing and potential targets for future microbiota-directed strategies aiming to promote piglet growth.

### Conclusions.

Weaning appears to be a key factor for the gut microbiota variation in the growing piglets. Transitions toward a mature gut microbiota enriched with fiber-degrading bacteria mostly complete upon weaning in piglets with better growth. Thus, postponing weaning age may favor the development of fiber-degrading gut bacteria, conferring the necessary capacity to digest and harvest solid postweaning feed. Alternatively, the potential of promoting the fast-growing-associated gut bacteria identified herein to nudge the microbiota into a mature state during weaning should be explored in future studies.

## MATERIALS AND METHODS

Four commercial farms in Finland were included in the study. A total of 670 stool samples were collected from 170 piglets from all four farms (farm 1 = 44 piglets, farm 2 piglets = 40 piglets, farm 3 = 44, and farm 4 = 42 piglets) at four sampling time points. Two fecal samples were collected during the suckling period (1st visit = 5 to 9 days of age, 2nd visit = 3 to 4 weeks of age) and two after weaning (3rd visit = 5 to 6 weeks of age, and 4th visit = 8 to 9 weeks of age). Additionally, for the comparative analysis with piglets, fecal samples were collected from the piglets’ mothers (40 sows from all four farms) at one time point (during the 1st visit). The general health conditions and treatments of all animals were recorded according to the regular management of the farms. One farm (farm 1; farm type 1) had enhanced environmental conditions compared with standard commercial farms. Sows farrowed freely in groups of five and piglets were also free to move in a common area with the other piglets and sows after 2 weeks of age, being able to suckle sow milk until 8 weeks of age (group lactation was 1 week shorter but essentially similar to what was described by van Nieuwamerongen et al. [49]).The rooms were provided with abundant straw bedding, and piglets were able to eat solid feed starting from around 2 weeks of age but were weaned completely (separated from the mother) only at 8 weeks of age. The other farms in the study (farms 2, 3, and 4; farm type 2) were conventional farms that provided limited amounts of bedding material. Piglet weaning on those farms was timed at the standard 4 weeks of age, therefore the length of the suckling period was half than in farm 1. Detailed information about the farms is given in Table S1 to 5.

### Sampling of animals.

We collected fecal samples from the mothers and from their female piglets only to avoid sex bias in the growth parameters. Female was the sex of choice because male piglets are surgically castrated at 3 to 4 days of age, which might cause stress and secondary infections at the time of our first sampling. Sows were balanced by parity. The first sampling was completed by collecting fecal samples from the selected sows (only one time point) and the female piglets from each farm at four sampling time points. Using sterile swabs, we collected fresh fecal samples from the rectum of piglets aged less than 8 weeks. We used hand gloves to collect fecal samples from older piglets and sows. We placed the samples in small sealable plastic bags (Minigrip, USA) and the samples were frozen immediately in dry ice for transportation to the laboratory, where they were stored at −70°C freezer until further processing.

At the first sampling, all the female piglets were individually weighed and ear tagged for further identification. At the second sampling, we selected at least four female piglets from each sow for sampling and, using the individual ear tags, followed them until the end with the third and fourth sampling. Dead, sick, or medicated piglets at any time were excluded from the study.

### Weighing of animal.

A digital hook scale (Pesola PHS040, China) and a fabric bag (handmade in Finland) were used for weighing of the piglets during visits 1 to 3. The correct functioning of the scale was tested before and during the weighing sessions using a standard test weight. At the final visit, because piglets exceeded 15 kg, for better practical accuracy, we used a cage scale (DV203E digital, Danvaegt, Hinnerup, Denmark) to record their weights.

### Average daily growth.

Individual ADG was used as a measure of growth. The ADG between sampling times 1 and 2 was calculated using the following equation:

$$\text{ADG}=\frac{\text{Weight during }2\text{nd sampling }-\text{ Weight during }1\text{st sampling}}{\text{Days between }1\text{st and }2\text{nd sampling}}$$

All the other ADG intervals were calculated in a similar way (Table 1). Piglets were classified according to their ADG within their farm. In each farm, piglets showing the highest overall ADG (≥third quartile; top 25%) and the lowest overall ADG (≤first quartile; bottom 25%) were assigned to the high-ADG and low-ADG group, respectively. For comparative analyses of the high- and low-ADG group, piglets with middle 50% ADG were cut out as a trade-off for the need to build sufficiently contrasting groups in terms of growth aptitude.

### Gut microbial DNA extraction and sequencing.

The Dneasy PowerSoil Pro Kit (Qiagen, ct. no. 47014, Hilden, Germany) was used to isolate microbial genomic DNA, taking 250 mg of fecal samples from all piglets, according to the manufacturer’s instructions (Qiagen, Hilden, Germany). Caution was taken to avoid potential contamination by including negative controls during DNA extraction and library preparation. The quantification of extracted DNA was measured with a Nanodrop spectrophotometer 2000 (Thermo Fisher Scientific, Waltham, MA, USA) to assess DNA purity. Library preparation and Illumina MiSeq sequencing of the hypervariable V3 to V4 regions of the 16S rRNA gene using primers 341F/785R were performed as previously described by Pereira et al. (50). The Illumina sequencing was done at the DNA Sequencing and Genomics Laboratory, Institute of Biotechnology, University of Helsinki.

### Bioinformatic and statistical analysis for microbiota profiling.

Sequence data processing and statistical analyses were performed with the statistical program R version 3.5.0 and RStudio version 0.99.903. *P*-values were corrected for multiple comparisons by using the Benjamini-Hochberg procedure (FDR). *P*-values and FDR-adjusted *P*-values < 0.05 were considered statistically significant. For differential abundance testing, an additional cut-off for the log_2_ fold change of ±1 (considered biologically significant) was set.

### Sequence data processing.

Out of the collected stool samples (N = 720), there were 710 samples (670 piglets and 40 sows) with corresponding sequence data. Demultiplexed reads after adaptor removal by cutadapt (51) were processed and merged using DADA2 (52), where *truncLenF* and *truncLenR* were set to 280 and 250, respectively, and reads with a number of expected errors higher than two were discarded, resulting in 17,586 ± 4,736 (mean ± SD) quality-controlled and chimera-checked reads per sample. Taxonomy was assigned to all identified amplicon sequence variants (ASVs) using a pretrained naive Bayes classifier implemented in DADA2 (the *assignTaxonomy* function with default settings, i.e., minBoot = 50) against the SILVA 132 reference database (53).

### Piglet microbiota analysis.

Microbiota α-diversity (observed richness and Shannon diversity index) was estimated using the *vegan* package (54). Overall microbiota structure was assessed using principal coordinate analysis (PCoA) based on β-diversity computed using the Bray-Curtis dissimilarity matrix, representing the compositional dissimilarity between samples or groups. Significant differences between groups were tested using nonparametric multivariate analysis of variance, PERMANOVA (55). The associations between continuous or categorical variables and β-diversity were calculated using the *envfit* function in the *vegan* package (56), and *P* values were determined using 999 permutations. The available microbiota covariates for analysis included sow parity, piglet oral vaccination against E. coli, postweaning feeding type, days of weaning age, piglet weight, average daily growth from sampling time 1 to 4 (overall), average daily growth from sampling time 2 to 4, average daily growth from sampling time 3 to 4, sow ID, and acid supplementation in drinking water.

To evaluate the impact of existing microbiota configuration on growth, ADG at sampling time 1 to 2, 2 to 3, 3 to 4, and overall ADG (sampling time 1 to 4) were predicted by the random forest regression (ntree (number of trees) = 10,001 and default mtry (number of random variables used in each tree) = *p*/3, where *p* is the number of input taxa; *R* package *random Forest*) using the relative abundances of bacterial genera at sampling time 1, 2, or 3 as input features. We then used repeated cross-validation (5-fold, 10 repetitions) of random forests in the *caret* package to evaluate the *R*^2^ of the gut microbiota to predict piglet growth. Associations between relative abundances of bacterial taxa and piglet ADG were further assessed using a linear mixed-effects model that accounted for the longitudinal structure of the data by treating the animal as a random effect. This model was further adjusted for sampling location (i.e., farm).

Dirichlet multinomial mixtures (DMM) clustering, an unsupervised clustering method known for its ability of handling sparse data (57), was used to identify groups of samples with similar microbial community structure. The optimal number of DMM clusters was determined by the lowest Laplace approximation score and cluster membership was assigned for all samples using the *DirichletMultinomial* package (58). The progression of samples through each DMM cluster was visualized using a customized *R* function available at https://github.com/aponsero/transition_plot_function. The maturity of the gut microbiota was estimated by computing a microbial-by-age z-score (MAZ) of each sample as described previously (59). Briefly, we started feeding a random forest model with a training set made of the microbial community composition of 30% randomly selected piglets that had a complete set of four samples. Once the model was trained, we used it to predict the age of all the samples. The age of the piglet predicted by this model was termed “microbiota age.” Finally, the z-score was computed using the following formula:

$$\text{MAZ}=\frac{\text{Microbial age}-\text{median microbial age of piglets at the same sampling time point}\;}{\text{Standard deviation of microbial age of piglets at the same sampling time point}}$$

Differentially abundant bacterial genera between sampling times or between ADG groups were identified with the *DESeq2* package (60) accounting for sampling location. *DESeq2* employs a generalized linear model of counts based on a negative binomial distribution, scaled by a normalization factor that accounts for differences in sequencing depth between samples. Significance testing was then assessed using the Wald test. Noncount variables (e.g., microbiota diversity, richness and MAZ) were analyzed with a Wilcoxon signed-rank test or paired *t* test for nonnormally distributed and normally distributed variables, respectively.

### Ethical permission.

This study was conducted according to the ethical guidelines of the University of Helsinki. The animal ethics permission for this study was granted by the Project Authorisation Board (formerly the Animal Experiment Board, ELLA) of the Regional State Administrative Agency, authorization number ESAVI/2325/04.10.07/2017 with modification ESAVI/17315/2020.

### Data availability.

The data sets generated in this study are available in the European Nucleotide Archive (ENA) repository, under accession no. PRJEB57171.
