# Supplementary material for: Impact of Intestinal Microbiota on Growth Performance of Suckling and Weaned Piglets
Source: Microbiol Spectr. 2023 Apr 6;11(3):e03744-22. doi: 10.1128/spectrum.03744-22 (PMC10269657; doi:10.1128/spectrum.03744-22)
Supplement: Supplemental file 1 — Tables S1 to S6. Download spectrum.03744-22-s0001.pdf, PDF file, 0.2 MB [file spectrum.03744-22-s0001.pdf]

Supplementary tables

Table S1. Basic information about the four farms included in this study. The commercial pig lines were produced by Figen Oy (Finnish Yorkshire and Landrace hybrids) or Finnpig Oy (Hyperprolific lines of Yorkshire, Landrace and Duroc hybrids).

Farm	Weaned piglets/sow*	Alive born piglets/sow*	Stillborn piglets/sow*	Mortality rate (%) before weaning*	Farrowing type	Sow breed
1.	10.4	12.56	0.98	15.3	Group free	Figen
2.	14.3	16.6	1.3	14.2	Crate	Finnpig
3.	12.0	14.4	1.4	16.5	Free	Figen
4.	12.5	14.8	1.1	9.1	Crate	Finnpig

*within one-year period from the last visit in the farm

Table S2. Sow and gilt numbers per farm, parity and feeding information.

Farm	n (sows/gilts)	Parity avg (min-max)	Sow feed during lactation
1	10 (5/5)	2 (1-6)	Complete feed (Emakko Pekoni 104 lyhyt rae, Suomen Rehu)
2	12 (9/3)	3 (1-6)	SF5579 IMETYS-PEKONI 1 PP GR 2F (until 2,5 days after farrowing) and SF5581 IMETYS-PEKONI 2 P GR 2F rest of time
3	11 (9/2)	3 (1-5)	A-Rehun imetys täysrehu + Pro2farm mure (Hankkija)
4	9 (7/2)*	3 (1-6)	Opti-Pekoni imetysrehu

*There were only eight litters from this farm. One gilt had to be euthanized – piglets were moved to another sow.

Table S3. Numbers of piglet samples by different samplings

Farm	1. Sampling	2. Sampling	3. Sampling	4. Sampling
1	44	42	44	44
2	40	39	40	40
3	44	44	42	44
4	42	42	41	42

Table S4. Age (d) by farm and visit, selected piglets

Farm	1. age avg (min-max)	2. age avg (min-max)	3. age avg (min-max)	4. age avg (min-max)
1	6 (5-9)	25 (22-29)	34 (31-38)	59 (56-63)
2	7 (6-7)	23 (22-23)	37 (36-37)	63 (62-63)
3	6 (5-9)	22 (21-25)	35 (34-38)	56 (55-59)
4	8 (7-9)	23 (22-24)	39 (38-40)	57 (56-58)

Table S5. Weight (kg) details by farm and visit for selected piglets

Farm	1. weight avg (min-max)	2. weight avg (min-max)	3. weight avg (min-max)	4. weight avg (min-max)
1	2.8 (1.7-3.8)	7.7 (4.9-11.8)	9.9 (6.5-13.5)	25 (17-33)
2	2.5 (1.3-4.2)	7.3 (4-9.8)	10.1 (6.7-14.6)	22.8 (15-32.8)
3	2.8 (1.7-4.1)	7.6 (3.7-10.5)	10.7 (5.8-14.7)	16.3 (9-22.5)
4	3.2 (1.9-4.8)	7.8 (5-11.6)	10 (6.4-13.6)	17.5 (9-24)

Table S6. Differentially abundant bacterial genera between Farm type 1 and 2. Nominally significant results are shown ($P < 0.05$). Log2 fold change was calculated using Farm type 1 as the reference group.

Genus	Log2 fold change	P	FDR-P
**Sampling time 1**			
Lactobacillus	-0.8339	0.038	0.122
Lachnoclostridium	-0.8182	0.047	0.157
Clostridium sensu stricto 1	2.5404	0.054	0.157
**Sampling time 2**			
Lactobacillus	-1.4253	0.045	0.237
*Rikenellaceae*_RC9 gut group	1.3457	0.047	0.237
**Sampling time 3**			
Clostridium sensu stricto 1	0.9893	0.023	0.086
*Rikenellaceae*_RC9 gut group	3.1223	0.037	0.102
Terrisporobacter	1.0206	0.037	0.102
*Ruminococcaceae*_UCG-005	4.3147	0.054	0.173
**Sampling time 4**			
Lactobacillus	-1.7320	0.013	0.061
Blautia	-1.6113	0.017	0.063
Clostridium sensu stricto 1	0.8258	0.021	0.081
*Ruminococcaceae*_UCG-008	0.7347	0.039	0.102
Faecalibacterium	-2.5460	0.039	0.102
Subdoligranulum	-0.8460	0.048	0.245
*Ruminococcaceae*_UCG-005	0.4276	0.052	0.248
**Sow**			
*Christensenellaceae*_R-7 group	4.3864	2E-06	2E-05
*Ruminococcaceae*_UCG-002	1.0794	2E-05	0.000
Treponema 2	3.2106	0.006	0.022
Romboutsia	2.1556	0.010	0.028
Turicibacter	4.1666	0.015	0.043
